# Supplementary material for: MetaRibo-Seq measures translation in microbiomes
Source: Nat Commun. 2020 Jun 29;11:3268. doi: 10.1038/s41467-020-17081-z (PMC7324362; doi:10.1038/s41467-020-17081-z)
Supplement: Supplementary file 10 — Supplementary Data 7 [file 41467_2020_17081_MOESM10_ESM.zip › File2/Confidence_High_Taxonomy/131806_out.krona.html]

Javascript must be enabled to view this page.

members
magnitude
magnitudeUnassigned
count
unassigned
taxon
rank

131806\_out

27

superkingdom
27
2

27
phylum
1239

27
class
186801

order
27
186802

4
family
541000

4
genus
216851

853

SRS050752\_contig\_number\_9986SRS143181\_contig\_number\_contig-100\_1218.90217SRS144753\_contig\_number\_contig-100\_2918.79129SRS148424\_contig\_number\_23455
species
4

1897045

SRS011084\_contig\_number\_11186SRS011134\_contig\_number\_contig-100\_1760.307597SRS014459\_contig\_number\_contig-100\_1718.224360SRS015431\_contig\_number\_contig-100\_5908.415651SRS016203\_contig\_number\_5505SRS018313\_contig\_number\_contig-100\_13311.13311SRS018817\_contig\_number\_23009SRS021484\_contig\_number\_31852SRS024132\_contig\_number\_contig-100\_1715.185243SRS024663\_contig\_number\_contig-100\_2011.95555SRS049446\_contig\_number\_3664SRS052697\_contig\_number\_contig-100\_44950.87227SRS056259\_contig\_number\_contig-100\_12003.130386SRS075773\_contig\_number\_contig-100\_2897.218613SRS098073\_contig\_number\_contig-100\_40255.40256SRS098571\_contig\_number\_60411SRS098881\_contig\_number\_contig-100\_1797.175924SRS101376\_contig\_number\_contig-100\_6599.151369SRS104400\_contig\_number\_59942SRS142503\_contig\_number\_contig-100\_1903.317958SRS143417\_contig\_number\_contig-100\_4178.181984SRS143876\_contig\_number\_28457SRS147614\_contig\_number\_6121
23
species
